# Caveolin-1 Deficiency in Macrophages Alleviates Carbon Tetra-Chloride-Induced Acute Liver Injury in Mice

**DOI:** 10.3390/ijms26104903

**Published:** 2025-05-20

**Authors:** Ruirui Li, Yixue Shu, Yulin Yan, Junyi Zhu, Zilu Cheng, Jie Zhang, Liming Zhu, Yanhua Qiao, Quan Sun

**Affiliations:** 1 Department of Laboratory Animal Science, School of Basic Medical Sciences, Capital Medical University, Beijing 100069, China; liruirui@mail.ccmu.edu.cn (R.L.); shuyixue@mail.ccmu.edu.cn (Y.S.); yanyulin985@163.com (Y.Y.); 2111158@mail.ccmu.edu.cn (J.Z.); cheng_zilu@mail.ccmu.edu.cn (Z.C.); 2 Laboratory Animal Center, Capital Medical University, Beijing 100069, China; angellena.student@sina.com (J.Z.); zhuliming@ccmu.edu.cn (L.Z.); jasmine422@ccmu.edu.cn (Y.Q.)

**Keywords:** Caveolin-1, bone marrow-derived macrophages, M2 polarization, hepatocellular necrosis and apoptosis, acute liver injury

## Abstract

Bone marrow-derived macrophages (BMMs) exhibit dynamic behavior and functional capabilities in response to specific microenvironmental stimuli. Recent investigations have proved that BMMs play crucial roles in promoting necrotic lesion resolution. Despite substantial advancements in understanding their activation and interaction with injured livers, researchers face challenges to develop effective treatments based on manipulating BMMs function. Caveolin-1 (Cav-1) is the major structural protein on the plasma membrane. We previously reported that Cav-1 knockout (KO) mice exhibited less functional damage and necrosis in carbon tetrachloride (CCl_4_)-induced liver injury. We hypothesize that the activation and recruitment of BMMs are involved in the resolution of necrotic lesions in Cav-1 KO mice. Wild-type (WT) and Cav-1 KO mice were injected with CCl_4_ (10% *v*/*v*) to induce acute liver injury model. Blood samples and hepatic tissues were harvested for serum alanine transaminase (ALT) activity assessment, histopathological examination through hematoxylin–eosin (H&E) staining, and BMMs subpopulation analysis via flow cytometry. Then, primary BMMs were isolated and cultured to investigate the effect of Cav-1 on BMMs polarization, migration, and activation of STAT3 signal pathway. Validation of hepatic macrophage depletion was induced by administrating clodronate liposomes (CLs), and BMMs reconstitution was evaluated by EGFP labelled BMMs. Following this, hepatic macrophages were depleted by CLs, BMMs were isolated from Cav-1 KO, and WT mice were cultured and administrated to evaluate the protective role of Cav-1-deleted BMMs on the resolution of hepatocellular necrosis and apoptosis in acute liver injury. The BMMs ratio significantly increased from 2.12% (1D), 4.38% (1W), and 5.38% (2W) in oil control mice to 7.17%, 14.90%, and 19.30% in CCl_4_-treated mice (*p* < 0.01 or *p* < 0.001). Concurrently, Cav-1 positive BMMs exhibited a marked elevation from 6.41% at 1D to 24.90% by 2W (*p* = 0.0228). Cav-1 KO exerted protective effects by reducing serum ALT by 26% (*p* = 0.0265) and necrotic areas by 28% (*p* = 0.0220) and enhancing BMMs infiltration by 60% (*p* = 0.0059). In vitro, Cav-1 KO BMMs showed a decrease in CD206 fluorescence intensity (*p* < 0.001), a time-dependent upregulation of arginase-1 mRNA (*p* < 0.05 or *p* < 0.01), a 1.22-fold increase in phosphorylated STAT3 (*p* = 0.0036), and impaired wound healing from 12 to 24 h (*p* < 0.001). The macrophage-depleting action in livers by CL injection persists for a minimum of 48 h. Administrated EGFP^+^ BMMs emerged as the predominant population following CL injection for a duration of 48 h. Following clodronate liposome-mediated hepatic macrophage depletion, the adoptive transfer of Cav-1 KO BMMs demonstrated therapeutic efficacy in CCl_4_-induced acute liver injury. In CCl_4_-induced acute liver injury, the adoptive transfer of Cav-1 KO BMMs reduced necrosis by 12.8% (*p* = 0.0105), apoptosis by 25.2% (*p* = 0.0127), doubled macrophages infiltration (*p* = 0.0269), and suppressed CXCL9/10 mRNA expression (*p* = 0.0044 or *p* = 0.0385). BMMs play a key role in the resolution of liver necrotic lesions in CCl_4_-induced acute liver injury. Cav-1 depletion attenuates hepatocellular necrosis and apoptosis by accelerating BMMs recruitment and M2 polarization. Cav-1 in macrophages may represent a potential therapeutic target for acute liver injury.

## 1. Introduction

Acute liver injury (ALI) is a severe and complex clinical condition characterized by high morbidity and mortality rates. Common etiological factors include hepatic ischemia-reperfusion (IR) injury, viral hepatitis, autoimmune disorders, and drug-induced hepatotoxicity [1]. Without timely diagnosis and intervention, ALI progresses rapidly to acute liver failure (ALF), a life-threatening condition associated with mortality rates exceeding 30% [2,3]. Identifying novel therapeutic targets and developing effective interventions to mitigate ALI progression, therefore, remains a crucial focus of hepatic research. Notably, hepatic necrotic lesions consistently manifest as a hallmark pathological feature across diverse ALI etiologies, making them a key target for therapeutic exploration [4].

Inflammation exerts a dual role in liver pathophysiology, acting as both a pathogenic driver and a protective mechanism. While it contributes to hepatic repair and fibrosis resolution, excessive or dysregulated inflammation can exacerbate tissue damage [5]. The hepatic macrophage population comprises two distinct subpopulations, bone marrow-derived macrophages (BMMs), originating from circulating monocytes, and resident Kupffer cells (KCs) that are embryonically established and self-renewing within the liver parenchyma. Both subsets exhibit functional plasticity, transitioning between proinflammatory mediators and proreparatory regulators depending on the injury phase and recovery dynamics [6,7]. During acute liver injury, the depletion of resident KCs triggers rapid infiltration of BMMs, which differentiate into KC-like cells to replenish the macrophage pool. This compensatory mechanism is critical for mitigating fibrosis and facilitating tissue repair [8]. Notably, in ConA-induced liver injury models, recruited BMMs demonstrate superior efficacy over KCs in resolving necrotic lesions, particularly when localized at necrotic borders [9]. This functional shift becomes indispensable when endogenous KCs are overwhelmed by severe injury [10]. Following tissue damage, infiltrating BMMs polarize toward an M2-like activation state, acquiring enhanced phagocytic capacity to clear necrotic debris and secrete proregenerative mediators, thereby accelerating the resolution of necrotic foci [11]. Under physiological homeostasis, KCs dominate the hepatic macrophage population. However, during acute injury, BMMs emerge as the predominant subset, orchestrating tissue recovery and representing promising therapeutic targets for liver disease intervention. Further investigation is required to elucidate the spatiotemporal regulation of macrophage phenotypes and their interplay during acute liver injury resolution [12,13].

Caveolin-1 (Cav-1), a 21–24 kDa integral membrane protein, functions as a key structural and regulatory component of caveolae. Through the oligomerization-driven organization of caveolar domains, Cav-1 scaffolds signaling complexes via its N-terminal scaffolding domain, directly modulating membrane-associated effectors, such as G-protein subunits, Src-family kinases, and growth factor receptors, to regulate cellular signal transduction [14]. Emerging evidence implicates Cav-1 in hepatic pathophysiology, with elevated expression observed in both clinical cirrhosis and experimental liver injury models [15,16]. Mechanistic studies link Cav-1 dysregulation to diverse liver pathologies spanning cholestasis, viral hepatitis, cirrhosis, and hepatocellular carcinoma [17]. Notably, Cav-1 deficiency has been shown to enhance cytoprotective autophagy by suppressing EGFR-PI3K-AKT-mTOR signaling, thereby inhibiting apoptosis during toxin-induced hepatotoxicity [18], attenuating acute liver injury via the blockade of the LPS-CD14-TLR4-NF-κB inflammatory axis [19], and regulating macrophage trafficking, with endothelial Cav-1 critically governing the extravasation of monocytes into inflamed tissues [20]. Our prior work further demonstrated that Cav-1 deficiency confers protection against CCl_4_-induced acute liver injury by skewing hepatic macrophages toward an anti-inflammatory M2 phenotype [21]. Despite these advances, the mechanistic interplay between Cav-1 and BMMs biology, particularly their infiltration dynamics, phenotypic polarization, and functional specialization during liver injury, remain an underexplored frontier requiring systematic investigation.

This study investigated the regulatory role of Cav-1 in BMMs recruitment and polarization during the resolution of necrotic lesions in carbon tetrachloride (CCl_4_)-induced acute liver injury. Key findings revealed that CCl_4_-challenged mice exhibited a significant upregulation of Cav-1 expression alongside enhanced BMMs infiltration. Genetic ablation of Cav-1 attenuated hepatocellular necrosis and apoptosis while augmenting BMMs recruitment to injury sites. In vitro assays demonstrated that Cav-1 deficiency promotes BMMs migration capacity and skews polarization toward an M2-reparative phenotype. Clodronate liposomes effectively induced the prolonged depletion of hepatic resident macrophages, with the suppressive effect maintained for a minimum of 48 h post-administration. The adoptive transfer of Cav-1-deficient BMMs into CL-pretreated mice enhanced necrotic lesion resolution, amplified BMMs recruitment, and reduced apoptotic indices compared to wild-type controls. These results establish that Cav-1 depletion accelerates necrotic resolution through dual mechanisms, enhancing BMMs migratory recruitment and driving M2-polarized repair programs. The macrophage-specific regulatory role of Cav-1 positions it as a promising therapeutic target for mitigating acute liver injury.

## 2. Results

### 2.1. Increased Infiltration of Cav-1 Positive BMMs in Acute Liver Injury

**Figure 1:**
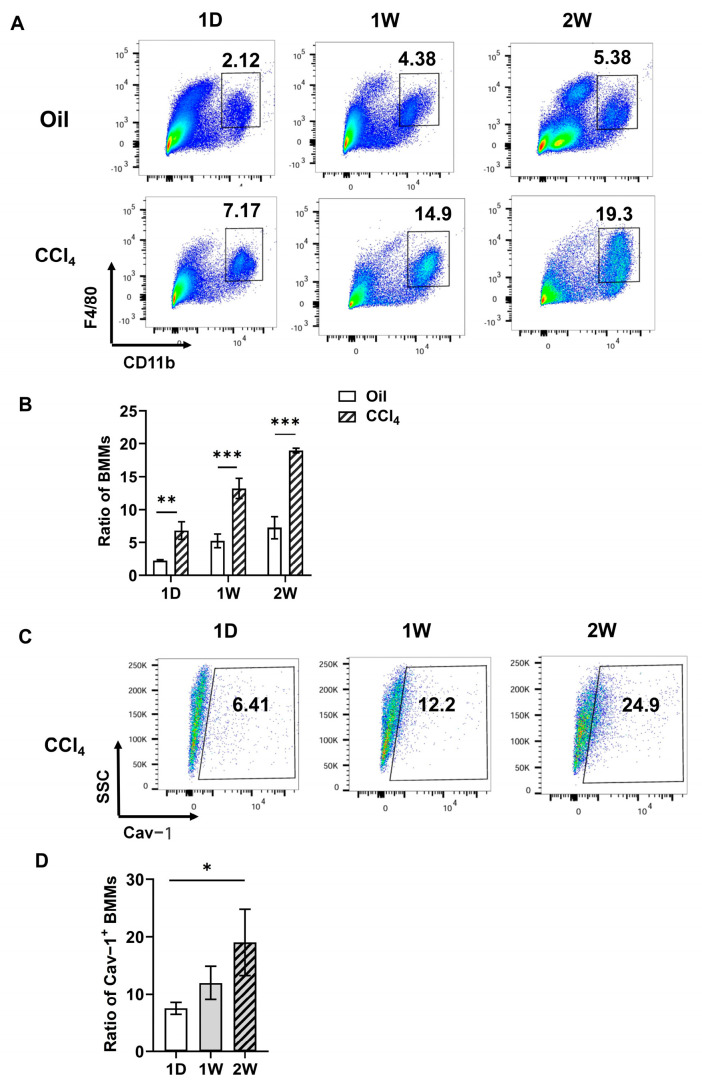
Temporal dynamics of Cav-1^+^ bone marrow-derived macrophages (BMMs) in CCl_4_-induced acute liver injury. Male C57BL/6J mice were challenged with CCl_4_ (10% *v*/*v*, IP) twice a week to induce acute liver injury. Liver non-parenchymal cells were isolated for flow cytometry analysis after 1 day, 1 week, and 2 weeks of treatment with CCl_4_. (**A**) Gating strategy for F4/80^+^CD11b^+^ BMMs populations in vehicle (oil) vs. CCl_4_-treated groups. (**B**) Quantification of BMMs infiltration showing Cav-1-KO vs. wild-type responses (two-way ANOVA). (**C**) Cav-1 expression profiling within BMMs (F4/80^+^CD11b^+^ gate). (**D**) Kinetic analysis of Cav-1^+^ BMMs expansion (one-way ANOVA with Tukey’s post hoc test). *n* = 5, * *p* < 0.05, ** *p* < 0.01, *** *p* < 0.01.

To elucidate the pathophysiological role of bone marrow-derived macrophages (BMMs) in acute hepatic injury, we established a carbon tetrachloride (CCl_4_)-induced liver damage model in male C57BL/6J mice. Animals received intraperitoneal injections of 10% (*v*/*v*) CCl_4_ solution twice weekly. Hepatic non-parenchymal cells were harvested at precisely defined intervals (1D, 1W, and 2W) post-injection for comprehensive immunophenotyping. Flow cytometric analysis revealed stable BMMs populations (F4/80^+^CD11b^+^) in vehicle control (oil-treated) mice across all timepoints (2.12% at 1D, 4.38% at 1W, 5.38% at 2W). Strikingly, Cav-1-deficient mice exhibited progressive BMMs expansion following CCl_4_ exposure, with percentages escalating from 7.17% (1D) to 14.90% (1W) and 19.30% (2W) (Figure 1A,B, ** *p* < 0.05 or *** *p* < 0.001 vs. control; detailed gating strategy in Supplementary Figure S1A).

To delineate the temporal dynamics of Cav-1-expressing BMMs, we performed longitudinal quantification using multiparametric flow cytometry. Post-CCl_4_ exposure, the proportion of Cav-1^+^ BMMs showed a progressive increase during the initial phase (1D to 1W). Notably, the F4/80^+^CD11b^+^ BMMs subpopulation expressing Cav-1 exhibited a significant expansion from 6.41% at 1D to 24.90% by 2W (*p* = 0.0228, Figure 1C,D). The F4/80^+^CD11b^+^Cav-1^+^ subpopulation exhibited a 3.9-fold enrichment during hepatic injury progression, suggesting phase-specific regulatory functions in injury pathogenesis. 

### 2.2. Cav-1 Deletion Reduced Liver Injury and Enhanced BMMs Infiltration

**Figure 2:**
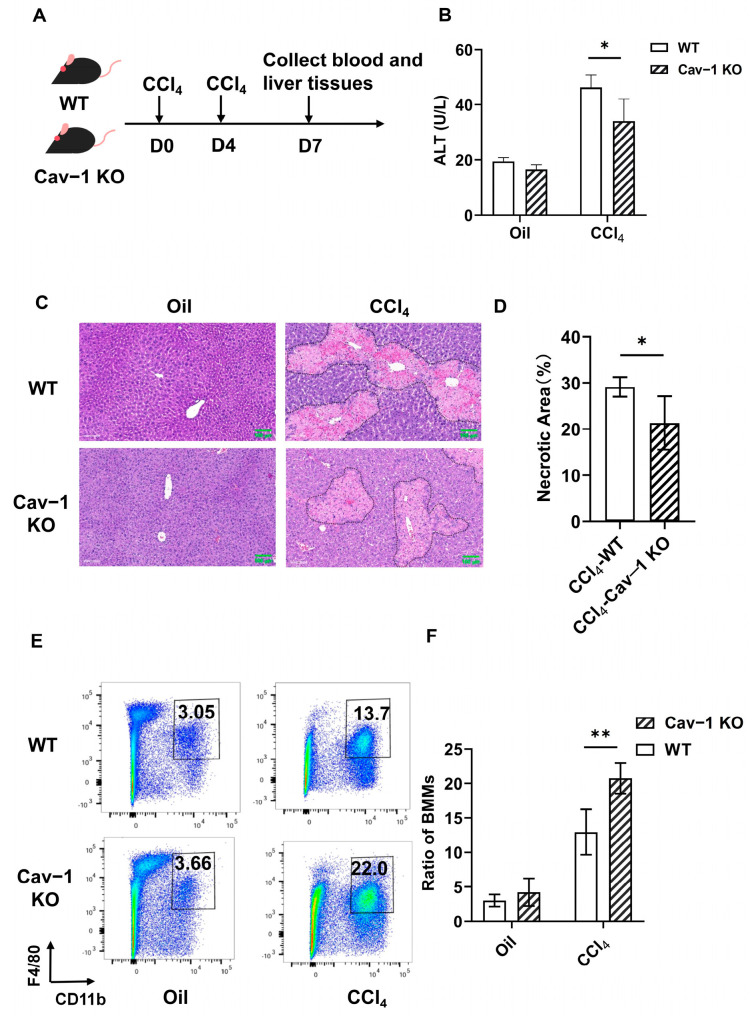
Cav-1 deletion enhances BMMs recruitment and attenuates CCl_4_-induced liver injury. (**A**) Experimental timeline. WT and Cav-1-KO mice received CCl_4_ injections on days 0 and 4 (with oil controls). Blood and liver tissues collected at day 7. (**B**) Serum ALT quantification (unpaired *t*-test). (**C**) H&E-stained liver sections. (**D**) Necrotic area quantification (unpaired *t*-test). (**E**) Representative plots for BMMs (F4/80^+^CD11b^+^) in WT and Cav-1 KO mice. (**F**) BMM proportions analyzed by two-way ANOVA. *n* = 5, Bar = 100 μm, * *p* < 0.05, ** *p* < 0.01 compared to WT mice.

Our prior work demonstrated that Cav-1 deficiency protects against CCl_4_-induced acute liver injury [22]. Given recent evidence highlighting BMMs as critical mediators of necrotic resolution in ConA-induced injury [14], we explored whether this protective mechanism involves BMMs recruitment. Employing Cav-1 knockout (KO) mice to model acute liver injury, we quantified BMMs infiltration through liver non-parenchymal cell analysis (Figure 2A). Cav-1-KO mice demonstrated significantly mitigated hepatic damage versus WT controls. Serum alanine aminotransferase (ALT) levels were decreased by 26% (from 46 to 34 U/L, *p* = 0.0265, Figure 2B) and necrotic areas were reduced from 29% to 21% (*p* = 0.0220, Figure 2C,D).

Prior evidence indicates BMMs surpass resident Kupffer cells in modulating hepatic injury [23]. To explore BMMs involvement in Cav-1-KO-mediated protection, we conducted flow cytometry to analyze F4/80^+^CD11b^+^ cells (BMMs) within the single-cell population. No significant BMMs proportion difference between WT (3.05%) and Cav-1-KO (3.66%) after oil treatment. Following CCl_4_ challenge, Cav-1 KO mice exhibited a 60% increase in BMMs infiltration (from 12.95% to 20.73%, *p* = 0.0059) compared to WT controls (Figure 2E,F). The gating strategy is listed in Supplementary Figure S1B. These data mechanistically link Cav-1 deficiency to enhanced BMMs recruitment during CCl_4_-induced necrosis resolution.

### 2.3. The Deficiency of Cav-1 Significantly Augmented M2-like Polarization and Migratory Capacity of BMMs In Vitro

**Figure 3:**
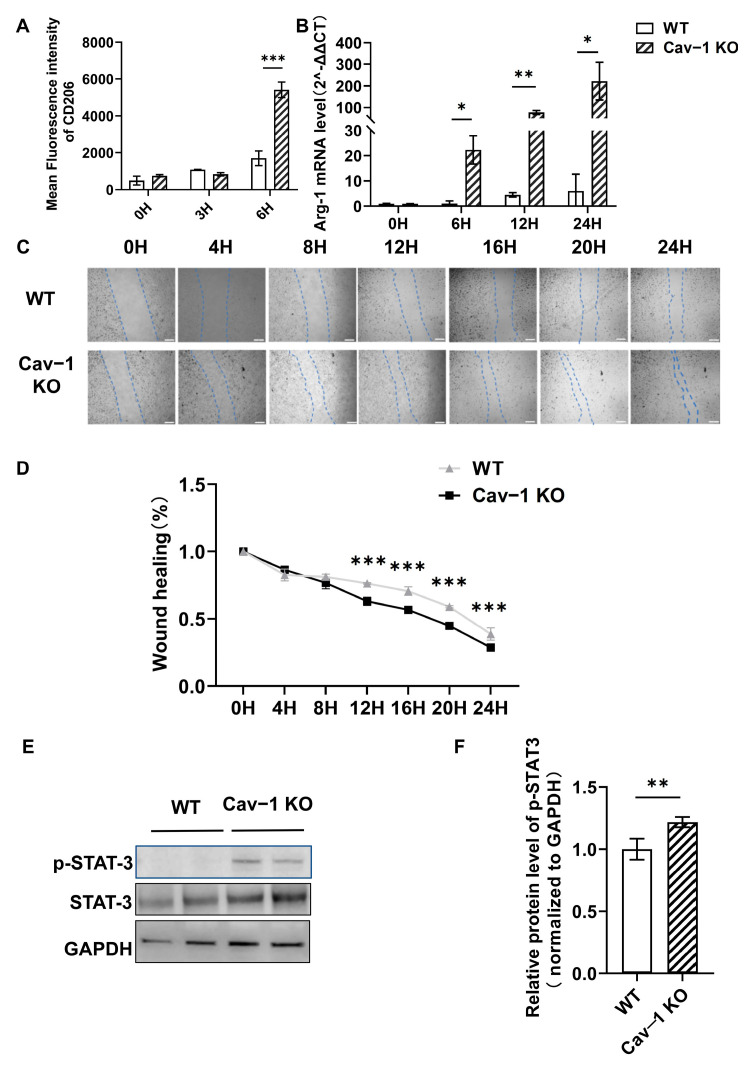
The deficiency of Cav-1 significantly augmented M2-like polarization and the migratory capacity of BMMs in vitro. (**A**) CD206^+^ macrophage quantification by flow cytometry at 0, 3, and 6 h post IL-4 stimulation (20 ng/mL). (**B**) Time-course Arg-1 mRNA expression in IL-4-treated BMMs (0–24 h). (**C**) Scratch wound healing assay of mature WT/Cav-1 KO BMMs (8 × 10^5^ cells/well) in serum-free medium. Dashed lines demarcate wound edges at indicated timepoints (0–24 h). Scale bar = 100 μm. (**D**) Wound closure rate quantification (remaining wound area/original area). (**E**) Representative Western blot of p-STAT3, total STAT3, and GAPDH. (**F**) Densitometric analysis of p-STAT3/GAPDH ratio. Data in (**A**,**B**,**D**,**F**) analyzed by two-way ANOVA (**A**,**B**,**D**) or Student’s *t*-test (**F**). Data represent mean ± SD. *n* = 3. Bar = 100 μm, * *p* < 0.05, ** *p* < 0.01, *** *p* < 0.001 vs. WT controls.

In acute liver injury, M1 macrophages mediate the inflammatory response by secreting proinflammatory cytokines that exacerbate tissue damage, whereas M2 macrophages contribute to tissue resolution through anti-inflammatory factor secretion and reparative functions [24]. To elucidate the regulatory role of Cav-1 in BMMs activation, we systematically investigated their functional characteristics. Following IL-4 stimulation (20 ng/mL, 6 h), flow cytometric analysis revealed a significant increase in CD206 (M2 macrophage marker) mean fluorescence intensity from 1695 to 5411 (*p* < 0.001) in Cav-1 KO BMMs compared to WTcontrols (Figure 3A). Complementary transcriptional profiling demonstrated that untreated Cav-1 KO and WT BMMs showed comparable baseline Arg-1 (M2 marker) mRNA levels. However, IL-4-treated Cav-1 KO BMMs exhibited a time-dependent upregulation of Arg-1 expression at 6, 12, and 24 h post-stimulation (*p* < 0.05 at 6, 24H, *p* < 0.01 at 12H; Figure 3B). We also analyzed Ym1 mRNA expression via qPCR (Supplementary Figure S1E). Cav-1 KO macrophages showed significantly increased Ym1 expression at 6 h post-IL-4 stimulation (*p* = 0.028).

Mechanistically, given the established role of JAK2/STAT3 signaling in macrophage polarization [25], we analyzed STAT3 activation. Western blot quantification showed 1.22-fold elevation (*p* = 0.0036) in phosphorylated STAT3 levels in Cav-1 KO BMMs (Figure 3E,F), suggesting enhanced pathway activation. 

The migratory ability of macrophages is fundamental to their overall functionality [26]. Functional assessment via a scratch wound healing assay revealed temporal differences in migration capacity. No significant variance was observed between genotypes within 8 h, but Cav-1 KO BMMs demonstrated markedly reduced wound closure percentages at 12–24 h (*p* < 0.001, Figure 3C,D), indicating augmented intrinsic migratory potential.

Collectively, these findings demonstrate that Cav-1 deficiency potentiates the M2-polarizing effect of IL-4 stimulation through STAT3 hyperactivation while concurrently enhancing the BMMs’ migratory capacity, suggesting a dual regulatory mechanism in macrophage functional modulation.

### 2.4. Validation of Hepatic Macrophage Depletion Followed by Adoptive BMMs Reconstitution

**Figure 4:**
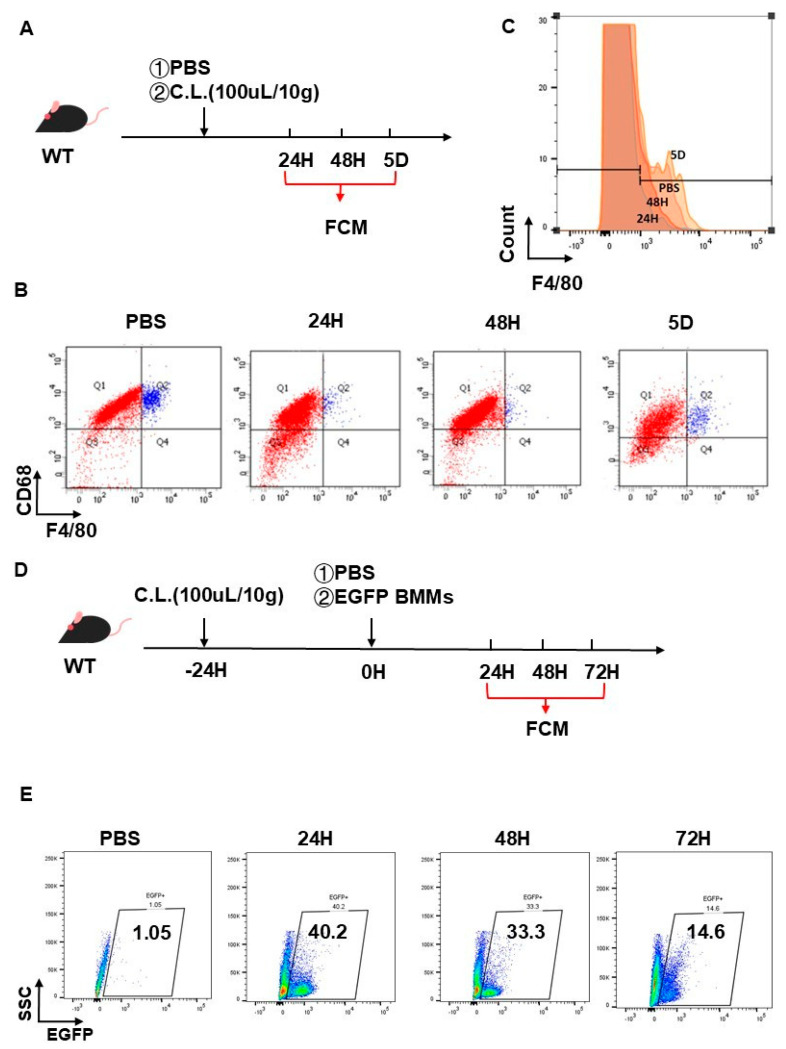
Validation of hepatic macrophage depletion followed by adoptive BMMs reconstitution. (**A**) Flow chart for study design. Resident hepatic macrophages were depleted by administrating clodronate liposomes (CLs). (**B**) Representative flow cytometry plots for F4/80^+^ macrophages in livers after CL treatment from 24 h to 5 days. (**C**) Histogram for efficiency of hepatic macrophage depletion by CLs. (**D**) Study design flowchart: administration of EGFP^+^ BMMs into mice after clodronate liposome treatment for 24 h. (**E**) Representative flow cytometry plots for EGFP^+^ BMMs in livers after administration from 24 to 72 h.

To mechanistically explore Cav-1’s regulatory role in BMMs infiltration during acute liver injury, we established a clodronate liposome (CL)-mediated hepatic macrophage depletion model coupled with adoptive BMMs transfer (Figure 4A). Intraperitoneal CL administration (100 μL/10 g BW) orchestrated a near-complete clearance of F4/80^+^ hepatic macrophages, virtually undetectable at 24 to 48 h post-injection as ascertained by flow cytometry. Notably, the hepatic F4/80^+^ macrophage population restored to levels equivalent to those observed in the PBS-injected control cohort at 5 days post-injection, which indicated that the macrophage-depleting action of CLs persists for a minimum of 48 h (Figure 4B,C).

Capitalizing on this temporal framework, EGFP^+^ BMMs (1 × 10^6^/mouse) were intravenously engrafted into CL-pretreated mice (24 h preconditioning) (Figure 4D). Flow cytometric tracking of hepatic non-parenchymal cells revealed peak engraftment efficiency at 24 h (40.2% EGFP^+^ cells), progressively declining to 33.3% and 14.6% at 48 h and 72 h post-transfer, respectively (Figure 4E). These biphasic kinetics establish a 48-h therapeutic window for studying exogenous BMMs dynamics in vivo.

### 2.5. Infusion of Cav-1-Deficient BMMs Attenuated Acute Liver Injury

**Figure 5:**
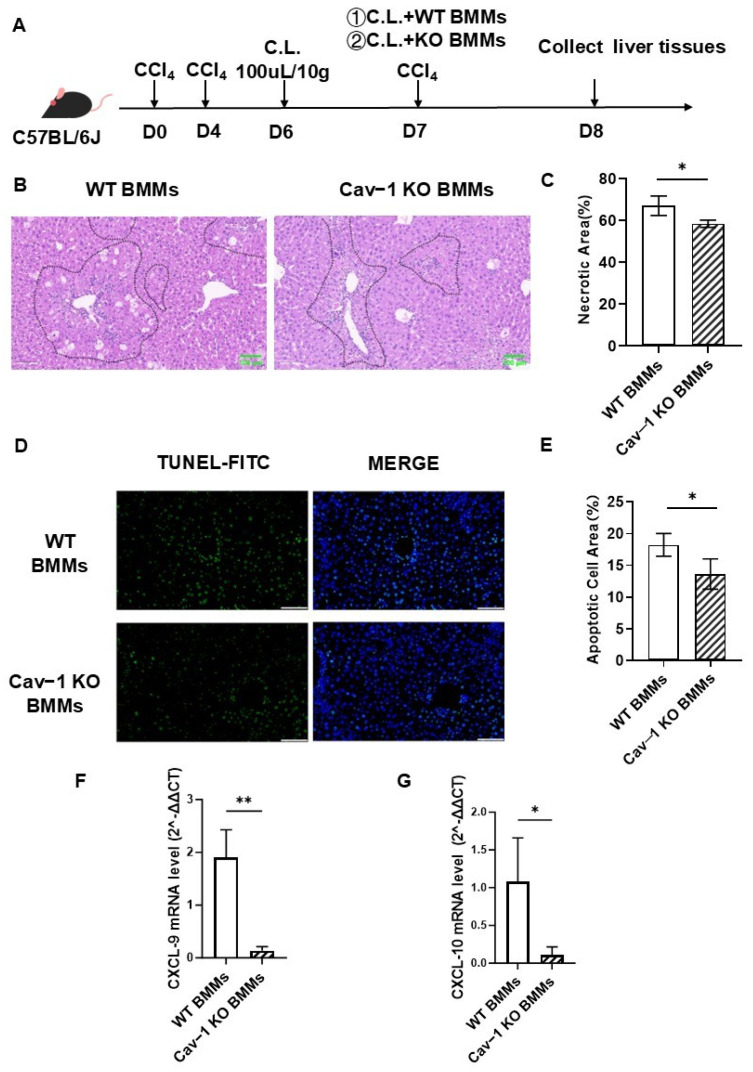
Cav-1-deficient BMMs ameliorate CCl_4_-induced acute liver injury. (**A**) Experimental timeline: Male C57BL/6J mice received intraperitoneal CCl4 (day 0, 4) followed by clodronate liposome depletion (day 6). Adoptive transfer of WT/Cav-1 KO BMMs (1 × 10^6^ cells via tail vein) was performed on day 7. (**B**) Hepatic necrosis analysis: representative H&E-stained sections comparing WT vs. Cav-1-deficient BMMs recipients. (**C**) Necrosis quantification: Cav-1-deficient BMMs reduced necrotic areas by 12.8% relative to controls (58.45% vs. 67.04%, * *p* < 0.05). (**D**) Apoptosis detection: TUNEL-stained liver sections demonstrating differential apoptotic signals between treatment groups. (**E**) Apoptosis quantification: Cav-1-deficient BMMs decreased apoptotic cell area by 25.2% (13.65% vs. 18.24%, * *p* < 0.05). (**F**,**G**) Chemokine modulation: significant downregulation of CXCL9 (** *p* < 0.05) and CXCL10 (* *p* < 0.01) mRNA levels in Cav-1-deficient BMMs recipients. Data analyzed by one-way ANOVA with Tukey’s post hoc test. *n* = 5, Bar = 100 μm, * *p* < 0.05, ** *p* < 0.01 vs. WT BMMs.

Building on these findings, we established an adoptive transfer system (Figure 5A) to assess the therapeutic potential of Cav-1-deficient BMMs in CCl_4_-induced acute liver injury. Male C57BL/6J mice received clodronate liposome pretreatment (24 h) followed by a tail vein injection of Cav-1 KO or WT BMMs (1 × 10^6^ cells/mouse). Liver injury parameters were evaluated 24 h post-transplantation.

Histopathological analysis revealed that Cav-1-deficient BMMs significantly reduced hepatic necrosis (58.45% vs. 67.04% in controls, *p* = 0.0105; Figure 5B,C) and apoptosis (13.65% vs. 18.24%, *p* = 0.0127; Figure 5D,E). Concurrently, we observed downregulated chemokine expression—C-X-C motif chemokine ligand 9 (CXCL9, *p* = 0.0044) and ligand 10 (CXCL10, *p* = 0.0385) (Figure 5F,G).

These results demonstrate that Cav-1 deficiency in BMMs confers hepatoprotective effects through dual mechanisms, attenuating parenchymal damage (12.8% reduction in necrosis; 25.2% decrease in apoptosis) and modulating chemokine-mediated immune cell recruitment.

### 2.6. Cav-1 Deficiency Enhanced BMMs Recruitment in Acute Liver Injury

**Figure 6:**
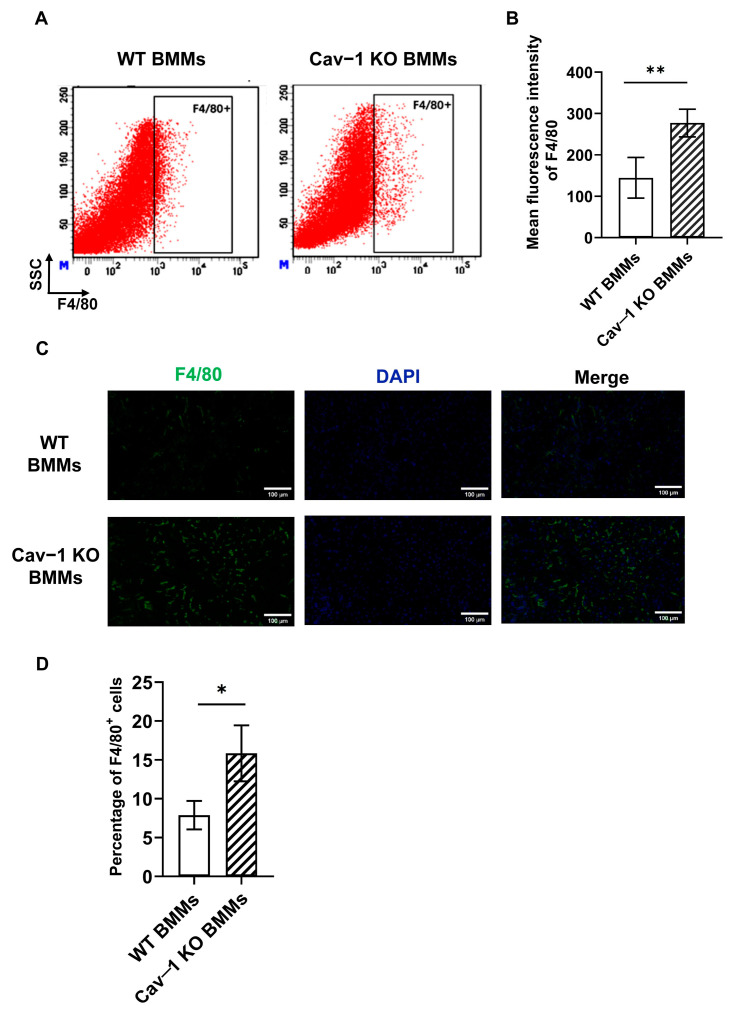
Cav-1 deficiency enhanced BMMs recruitment in acute liver injury. (**A**) Representative flow cytometry plots for F4/80^+^ macrophages in damaged livers after Cav-1 KO or WT BMMs administration for 24 h. (**B**) Analysis for mean fluorescence intensity of F4/80^+^ macrophage populations in damages livers. (**C**) Representative immunofluorescence images for F4/80^+^ macrophages (green) in damaged livers. (**D**) Quantification of the percentage of F4/80^+^ cells in livers of Cav-1 KO or WT BMMs-treated animals. Data were analyzed by one-way ANOVA for multiple comparisons and Tukey’s post hoc test for the significance of difference analysis between Cav-1 KO BMMs and WT BMMs groups. *n* = 5, Bar = 100 μm, * *p* < 0.05, ** *p* < 0.01.

It has been proved that BMMs were more efficient in clearing necrotic tissue, thereby facilitating lesion resolution [8,9]. Therefore, we evaluated whether the protective role of Cav-1-deficient BMMs in acute liver injury was related to enhanced BMMs recruitment. Because hepatic macrophages were deleted by clodronate liposomes, infiltrated F4/80^+^ macrophages were derived from administrated Cav-1 KO or WT BMMs. In our subsequent analyses, flow cytometry was utilized to quantitatively evaluate the hepatic infiltration of BMMs. We observed a significant increase in the mean fluorescence intensity of F4/80 (macrophage specific marker) in the Cav-1 KO BMMs-treated livers (Figure 6A,B).

To validate the flow cytometry data and ascertain the spatial distribution of BMMs within the damaged livers, immunofluorescence staining was also performed. As shown in Figure 6C,D, F4/80 positive cells were significantly increased in the damaged livers of Cav-1 KO BMMs-treated animals compared to the WT BMMs control.

We conducted an immunofluorescence analysis of liver tissues. The results reveal a significantly elevated proportion of F4/80^+^CD206^+^ macrophages (established M2 markers) in mice receiving Cav-1 KO BMMs compared to WT BMMs-treated controls. Representative images showing F4/80^+^CD206^+^ macrophage populations are provided in Supplementary Figure S1G. These in vivo findings corroborate our previous in vitro observations of enhanced M2 polarization in Cav-1-deficient macrophages under IL-4 stimulation.

Flow cytometric analysis using F4/80 and CD11b markers were conducted in clodronate-treated mice. The data confirm the near-complete depletion of resident KCs (F4/80^+^CD11b^−^), verifying that the observed effects are mediated by adoptively transferred BMMs (F4/80^+^CD11b^+^). Representative images showing F4/80^+^CD11b^−^ KCs populations are provided in Supplementary Figure S1F.

These collective findings indicated that Cav-1 deficiency increased the recruitment and M2 polarization of BMMs in damaged liver tissues, which may explain the reason why the infusion of Cav-1-deficient BMMs was effective to attenuate hepatocellular necrosis and apoptosis in acute liver injury.

## 3. Discussion

Liver necrotic lesions are a common pathological feature observed in various human hepatic disorders. However, the cellular mechanisms underlying necrotic lesion resolution remain complex and exhibit heterogeneity across different etiologies of acute liver injury. Emerging evidence highlights bone marrow-derived macrophages (BMMs) as critical mediators in resolving necrotic lesions during liver injury [12,14]. Caveolin-1 (Cav-1), the principal structural component of plasma membrane caveolae, functions as a signaling hub through interactions with diverse signaling molecules [27]. In this study, we demonstrated elevated BMMs recruitment coupled with upregulated Cav-1 expression in CCl_4_-induced acute liver injury. Genetic ablation of Cav-1 enhanced BMMs infiltration while mitigating hepatocellular necrosis. Mechanistically, Cav-1 was identified as a pivotal regulator orchestrating both BMMs activation and recruitment. In vitro experiments revealed that Cav-1 knockout (KO) potentiated BMMs migratory capacity and promoted M2 polarization. Notably, the adoptive transfer of Cav-1-deficient BMMs ameliorated hepatocyte necrosis and apoptosis, concomitant with augmented BMMs recruitment and downregulated CXCL-9/10 expression. These findings collectively establish Cav-1 as a master regulator of BMMs dynamics during necrotic lesion resolution in acute liver injury.

The establishment of coordinated inflammatory resolution mechanisms following immune activation is critical for maintaining hepatic homeostasis during pathogen invasion or tissue damage [28]. Hepatic macrophages exist as two principal subsets, BMMs and Kupffer cells (KCs), with distinct functional roles in injury responses. Intriguingly, KCs populations undergo rapid functional exhaustion during liver injury, prompting their replacement by recruited BMMs. Spatial mapping in murine models of concanavalin A (ConA)-induced liver injury revealed that BMMs form perinecrotic clusters that coordinate multicellular regenerative processes through intercellular cross-talk [14]. Furthermore, lineage tracing studies demonstrate that BMMs exhibit remarkable plasticity, differentiating into monocyte-derived KCs (MoKCs) upon KC depletion, with MoKC-reconstituted livers showing significant attenuation of functional impairment, necrotic progression, and fibrotic remodeling [14,28]. These findings collectively position BMMs as both key mediators of hepatic repair and viable therapeutic targets. Global Cav-1 deficiency was associated with exacerbated macrophage infiltration, whereas the administration of Cavtratin, a cell-permeable Cav-1 scaffolding domain mimetic, suppressed macrophage survival and migratory capacity [29]. This regulatory dichotomy extends beyond hepatic systems, as evidenced by breast cancer studies showing that macrophage migration inhibitory factor (MIF)-mediated Cav-1 phosphorylation modulates CD11b^+^ immune cell recruitment [30]. Consistent with these mechanistic insights, we observed a significant expansion of F4/80^+^CD11b^+^ BMMs populations in Cav-1 KO livers post-injury. In vitro scratch wound healing assays quantitatively demonstrated enhanced migratory capacity in Cav-1-deficient BMMs. Crucially, the adoptive transfer of Cav-1 KO BMMs attenuated acute hepatocellular necrosis and apoptosis, a therapeutic effect associated with amplified BMMs recruitment and the concomitant downregulation of CXCL-9/10 chemokine expression. This regulatory axis orchestrates the spatial–temporal recruitment of reparative macrophages, thereby facilitating necrotic lesion resolution in acute liver injury.

Macrophages demonstrate remarkable functional plasticity, dynamically adapting their phenotype in response to microenvironmental cues [31]. These cells are broadly categorized into classically activated (M1) macrophages, which secrete proinflammatory mediators, and alternatively activated (M2) macrophages, characterized by enhanced phagocytic capacity and anti-inflammatory functions [32,33]. Emerging evidence implicates Caveolin-1 (Cav-1) as a regulator of macrophage polarization. Clinical studies indicate elevated serum Cav-1 levels in non-alcoholic fatty liver disease (NAFLD) patients, accompanied by cell-type-specific expression patterns, hepatocyte Cav-1 downregulation contrasts with macrophage-specific upregulation in vivo. Cav-1-expressing CD68^+^CD163^+^ M2 macrophages exacerbate NAFLD progression by disrupting iron homeostasis [34]. Besides, myocardial infarction models demonstrate that Cav-1 reduction correlates with increased M2 macrophage infiltration within infarct zones [35]. In vitro analyses further confirm that Cav-1-deficient macrophages exhibit amplified IL-4-induced M2 polarization [36]. Our data align with these observations, showing that Cav-1 KO BMMs display a significantly upregulated expression of M2-associated markers CD206 and arginase-1 compared to wild-type controls [37]. This phenotypic shift coincides with enhanced STAT3 phosphorylation, a critical mediator of macrophage polarization [25]. Mechanistically, Cav-1 deficiency likely promotes BMMs M2 polarization through the JAK2/STAT3 pathway activation, consistent with prior findings in rat atrial fibroblasts [38].

M1 macrophages secrete proinflammatory mediators, including C-X-C motif chemokine ligand 9 (CXCL9) and 10 (CXCL10), which coordinate effector cell recruitment to injury sites and correlate with clinical disease progression [39]. In HBV-induced liver injury, serum CXCL9/CXCL10 levels demonstrate significant elevation and strong positive correlations with hepatic transaminases, establishing their utility as prognostic biomarkers [40]. Our experimental data align with these clinical observations, demonstrating that Cav-1 KO BMMs-treated animals exhibit a significant reduction in CXCL9 and CXCL10 levels. This evidence supports the mechanistic hypothesis that Cav-1 deficiency mitigates acute liver injury through CXCL9/10 downregulation in macrophages. Further research is required to evaluate whether Cav-1 deficiency in macrophages leads to a decreased expression of CXCL9/10, responsible for the recruitment of T/NKT cells infiltration into the liver as previously reported [18].

Emerging evidence highlights bone marrow-derived macrophages (BMMs) as promising candidates for cell-based therapies in chronic liver diseases. Endogenous macrophage populations demonstrate therapeutic efficacy through targeted migration to fibrotic lesions and the subsequent promotion of extracellular matrix remodeling [41,42]. Notably, both unprimed (M0) and classically activated (M1) BMMs attenuate hepatic fibrosis, with M1-polarized cells exhibiting superior matrix metalloproteinase activity and collagen degradation capacity [43]. Furthermore, colony-stimulating factor-1-induced BMMs were reported to have a therapeutic effect on CCl_4_-induced cirrhosis in mice [44]. In contrast to well-characterized chronic applications, BMMs therapeutic mechanisms in acute hepatic injury remain underexplored. Recent studies indicated that infiltrating BMMs are essential in facilitating the resolution of necrotic lesions associated with liver injury [12,14]. Our investigation reveals that BMMs achieve hepatic accumulation within 24 h post-administration, which emerged as the predominant population following clodronate liposome injection for a duration of 48 h. We further proved that Cav-1 deficiency enhanced BMMs recruitment, which was involved in alleviated hepatocellular necrosis and apoptosis in damaged livers. The enhanced therapeutic efficacy of Cav-1-KO BMMs establishes them as promising candidates for cell therapy in acute liver injury.

Our findings highlight the crucial role of Cav-1 in regulating BMMs activation and recruitment as well as modulating the expression of chemokines involved in necrotic lesion resolution, which may provide a new target for treating acute liver injury.

Limitations of the study: Image J Fiji was utilized for image quantification. However, version-specific plugin compatibility may affect reproducibility in cross-platform analyses. While our results are preliminary, they provide mechanistic insights into macrophage-specific Cav-1 functions. We fully agree that future validation using myeloid-specific Cav-1 knockout mice would strengthen the conclusions.

## 4. Materials and Methods

### 4.1. Mouse Strain

Cav-1 KO mice (Cav-1^−/−^, STOCK Cav-1^tm1Mls/J^) and WT mice (C57BL/6J) were purchased from Jackson Laboratory. EGFP mice were obtained from Cyagen Laboratory. The experimental protocol was approved by the Animal Experiments and Experimental Animal Welfare Committee of Capital Medical University (Approval Number: AEEI-2022-270, 2022). All animal studies were performed in accordance with the Guidelines of the Animal Experiments and Experimental Animals Management Committee of Capital Medical University.

### 4.2. Acute Liver Injury Model

In this study, male Cav-1 KO mice and WT mice, 8–10 weeks old and weighing 25–28 g, were used. Animals were randomly allocated into experimental groups via computer-generated sequence, with group sizes (*n* = 5) determined by power analysis (α = 0.05, β = 0.8) based on pilot studies. An acute liver injury model was successfully induced through twice-weekly intraperitoneal injections of carbon tetrachloride (CCl_4_) solution. The CCl_4_ was diluted in olive oil at a 1:9 volume ratio (*v*/*v*) and administered at a standardized dosage of 10 microliters per gram of body weight (10 μL/g bw). Control mice were injected with olive oil only. Mice were sacrificed by CO_2_ exposure under the guidelines of designed experimental approaches.

### 4.3. BMMs Isolation and Culture

As described previously, bone marrow-derived macrophages (BMMs) were isolated and cultured for 7 days. WT, Cav-1 KO, and EGFP mice were euthanized, following which both the femur and tibia were excised. Bone marrow was then extracted from the femur and tibia and collected into ice-cold PBS using a 23G needle. After centrifugation and filtration with a 70 μm cell strainer (corning falcon, 352350), cells were resuspended by DMEM with 10 ng/mL M-CSF (C600234-0010, Sangon Biotech, Shanghai, China) for BMMs culture. Matured BMMs were collected on day 7 for further testing. All cellular experiments were performed in triplicate independent experiments using cells from three separate WT or Cav-1 KO animals (*n* = 3).

### 4.4. Flow Cytometric Analysis of Liver Macrophages

The liver was perfused with 0.04% Collagenase IV-PBS for digestion. The digested liver tissue was gently dissociated using forceps and filtered through a 70 μm cell strainer. The cell suspension was centrifuged at 60× *g* for 2 min, and the pellet was discarded. The supernatant containing the non-precipitated cells was collected and further centrifuged at 700× *g* for 6 min. After discarding the supernatant, the cell pellet was collected for subsequent experiments. Appropriately collected cells were resuspended in ice-cold PBS supplemented with 10% FBS before treatment with fluoresce-conjugated antibodies. FITC-F4/80 (Abcam, Cambridge, UK, ab60343), PE-CD68 (eBioscience, 2252601), Alexa Fluor647-Caveolin-1 (Cell Signaling Technology, Danvers, MA, USA, 3144S), APC-CD11b (Abcam, Cambridge, UK, ab25482), PEcy7-CD11b (Abcam, Cambridge, UK, ab218786), and PE-CD206 (Santa Cruz Biotechnology, Paso Robles, CA, USA, sc-58986) were used in flow cytometry analysis at recommended concentrations. Following a 30-min incubation in the dark, cell suspensions were washed three times with ice-cold PBS containing 10% FBS. Flow cytometry was performed on BD LSRFortessa and analyzed using BD FACSDiva 7.0 and Flowjo-V10.58986, which were used in flow cytometry analysis at recommended concentrations. The ratio of BMMs in hepatic single-cell suspensions was quantified by flow cytometric analysis of F4/80^+^CD11b^+^ double-positive cells. To determine the ratio of Cav-1^+^ BMMs, we calculated the percentage of Cav-1^+^ cells within the total F4/80^+^CD11b^+^ macrophage population using dual parameter gating strategies. The fluorescence intensity of F4/80 and CD206 were normalized against isotype controls using FlowJo v10.8.

### 4.5. RT-qPCR

Total RNA from liver tissues or BMMs was extracted by Total RNA Extraction Kit according to the manufacturer’s instructions. Primers were designed as follows: Mouse *β-actin*: forward; 5′-TCT GGC ACC ACA CCT TCT AC-3′; reverse; 5′-TAC GAC CAG AGG CAT ACA GG-3′. Mouse *Arg-1*: forward; 5′-AAG AAT GGA AGA GTC AGT GTG G-3′; reverse; 5′-GGG AGT GTT GAT GTC AGT GTG-3′. Mouse *CXCL-9*: forward; 5′-ATCTTCCTGGAGCAGTGTGG-3′; reverse; 5′-AGTCCGGATCTAGGCAGGTT-3′. Mouse *CXCL-10:* forward; 5′-GTGAGAATGAGGGCCATAGG-3′; reverse; 5′-GGCTAAACGCTTTCATTAAATTC-3′. All real-time RT-PCR reactions were performed in Quant Studio 3D digital PCR System (Applied Biosystem Life Technologies, Shanghai, China). Expression levels of Arg-1, CXCL-9, and CXCL-10 were normalized to β-actin (internal control) and calculated using the 2^−ΔΔCT^ method. 

### 4.6. BMMs Polarization Analysis

After 7 days of culture, Cav-1 KO or WT BMMs (8 × 10^5^ cells/well) were washed with sterilized PBS three times, and the culture medium was replaced with complete DMEM containing 20 ng/mL recombination mouse IL-4. Cells were collected at 0, 3, 6, 12, and 24 h following induction, treated with 0.2% EDTA for dissociation, and then resuspended in ice-cold PBS. Flow cytometry assay was then performed with FITC-F4/80 (Abcam, Cambridge, UK, ab60343) and CD206 (Santa Cruz, CA, USA, sc-58986) for M2 macrophage polarization. For measuring M2 polarization of macrophages, mean fluorescence intensity of CD206 was calculated. Also, we utilized quantitative PCR (QPCR) to measure the expression levels of arginase-1 (Arg-1), a canonical marker of M2 macrophages.

### 4.7. Cell Scratch Assay

The cell scratch assay was conducted to evaluate cell migration. Matured WT or Cav-1 KO BMMs (8 × 10^5^ cells/well) were cultured in serum-free medium to assess migration independent of proliferation. Wound healing (wound length/origin wound length) was monitored at 0, 4, 8, 12, 16, 20, and 48 h under a microscope and calculated by Image J v2.9.0 software. The wound region was manually outlined to calculate the pixel area. For each time point, the mean percentage of wound healing was calculated from three independent experiments.

### 4.8. H&E Staining

For the histological assessment of hepatocellular necrosis, a total of 5 animals were utilized for each experimental group. Liver tissues were fixed in 4% paraformaldehyde for histopathological examination. Paraffin-embedded liver tissues were sliced into 5-μm-thick sections and stained with H&E to facilitate the morphological examination of liver tissue necrosis. Bright-field images were obtained via the Pannoramic Scan auto-scan system and analyzed with Image J. The results were shown as necrotic area (area of the necrotic hepatocyte cells/area of the whole field), which was measured in at least 5 random fields per section.

### 4.9. TUNEL Staining

After 5-μm-thick liver sections were prepared, TUNEL staining was performed to analyze hepatocyte apoptosis according to the manufacturer’s instruction (Roche Diagnostics GmbH, Mannheim, Germany). Liver sections were incubated in 50 mL of TUNEL reaction mixture for 60 min at 37 °C. Samples were observed under a fluorescence microscope at a range of 515–565 nm (green). The results were shown as apoptotic cell area (number of TUNEL-positive cells /total number of DAPI-stained cells), which was measured in at least 5 random fields per section.

### 4.10. Measurement of ALT

Blood samples obtained from different groups of mice were properly treated to acquire high-quality serum. Serum alanine transaminase (ALT) was measured via biochemistry analyzers (TBA40FR, TOSHIBA, Tokyo, Japan).

### 4.11. Hepatic Macrophages Depletion and BMMs Reconstitution

In order to evaluate the availability of resident hepatic macrophage depletion and BMMs reconstitution, a mice model was induced by administrating clodronate liposomes (CL, 100 µL/10 g body weight) to deplete liver macrophages. Control animals received the same volume of PBS. Following intraperitoneal administration of CLs or PBS, primary non-parenchymal cells were isolated and F4/80^+^ macrophages were detected by flow cytometry from 24 h to 5 days post CL injection.

Based on above results, EGFP^+^ BMMs were then isolated and cultured from EGFP mice. We administrated EGFP^+^ BMMs (1 × 10^6^/mice) or PBS into mice by tail vein, which were treated with CLs 24 h before administration. Then, 24 to 72 h post-injection of EGFP^+^ BMMs, primary non-parenchymal cells were isolated to identify the efficiency of BMMs infiltration by flow cytometry.

### 4.12. BMMs Infusion

An acute liver injury model was established by intraperitoneal injection with a mixture of CCl_4_ and olive oil (1:9 *v*/*v*) at a dose of 10 µL/g of body weight on day 0 and 4. After 24 h post-injection with clodronate liposome injection (on day 6), isolated primary WT or Cav-1 KO BMMs were administrated by tail vein at a concentration of 1 × 10^6^ cells per animal (on day 7). Mice were euthanized 24 h after BMMs administration and liver tissues were collected (on day 8) for further research.

### 4.13. Western Blot Analysis

Protein extracts from WT or Cav-1 KO BMMs were separated by SDS-PAGE and transferred to PVDF membranes. Membranes were blocked with 5% milk in TBST for 1 h at room temperature to minimize non-specific binding. They were then incubated with anti-STAT3 antibody (1:1000, Abcam, Cambridge, UK, ab68153) and anti-phospho-STAT3(Tyr705) antibody (1:1000, Cell Signaling Technology, 9138S) overnight at 4 °C, followed by incubation with an HRP-conjugated secondary antibody (goat anti-rabbit antibody (1:5000, ZB-2301); goat anti-mouse antibody (1:5000, ZB-2305)) for 1 h at room temperature. Specific protein bands were detected using an ECL system and quantified by densitometry after normalization to GAPDH (Thermo Fisher Scientific, Waltham, MA, USA, MA5-15738) as a loading control. All procedures were performed in accordance with standard Western blot techniques, ensuring the accuracy and reproducibility of protein detection. Western blot band intensities were quantified using Image J v2.9.0 software. Images were converted to 8-bit grayscale, and background subtraction was applied. Rectangular ROIs were used to measure the intensity of target and internal control bands. The relative expression of p-STAT3 was normalized to GAPDH as an internal control. In our experiments, each group was represented by a minimum of three replicate samples, and each experiment was independently conducted on three separate occasions to ensure the reproducibility and robustness of the results.

### 4.14. Immunofluorescence Staining

Paraffin-embedded liver tissues were sectioned at 5-μm-thick by a microtome (Leica, Bensheim, Germany, CM1950). Then, the sections were deparaffinized and rehydrated through a series of xylene and graded ethanol washes. Antigen retrieval was performed by heating the sections in citrate buffer (pH 6.0) for 20 min. Endogenous peroxidase activity was quenched with 3% H_2_O_2_ for 10 min, followed by blocking with 5% bovine serum albumin (BSA) in phosphate-buffered saline (PBS) for 1 h at room temperature to minimize non-specific binding. Primary antibodies, anti-F4/80 antibody (1:1000, Abcam, ab300421) were applied to the sections. The sections were incubated with the primary antibodies in PBS containing 1% BSA overnight at 4 °C. After washing with PBS, the sections were incubated with the following donkey anti-rabbit IgG Alexa Flour Plus 488 (Thermo Fisher, A32790) for 1 h at room temperature. Nuclei were counterstained with DAPI (Beyotime, Shanghai, China, C1006) to visualize cellular morphology. Images were captured and analyzed to determine the expression and localization of the target proteins within the tissue sections. The results were shown as the percentage of F4/80 positive cells (mean gray value of F4/80 positive cells/total number of DAPI-stained cells), which was measured in at least 5 random fields per section.

### 4.15. Statistics

Statistical analyses were performed with GraphPad Prism version 8.0 software. Bar graphs show the mean ± SD, as indicated in each legend. Differences between groups were examined by *t*-test or one-way ANOVA for multiple comparisons and Tukey’s post hoc test or two-way ANOVA for multiple comparisons test. Images were treated using Image Scope. A value of *p* < 0.05 was considered statistically significant.

## 5. Conclusions

Our research collectively underscores the crucial function of BMMs in the resolution of necrotic lesions in CCl_4_-induced acute liver injury. Depletion of Cav-1 promotes recruitment and M2 polarization of BMMs as well as a reduced expression of CXCL9 and CXCL10, which may enhance the resolution of liver necrotic lesions. Therefore, Cav-1 deficient BMMs may exhibit potent functions in promoting the resolution of necrotic lesions in acute liver injury.

## Data Availability

Data in this study can be made available upon request to the corresponding author, Quan Sun (sunquan@ccmu.edu.cn).

## Supplementary Materials

The following supporting information can be downloaded at: https://www.mdpi.com/article/10.3390/ijms26104903/s1.
