# Supplementary material for: Caveolin-1 Deficiency in Macrophages Alleviates Carbon Tetra-Chloride-Induced Acute Liver Injury in Mice
Source: Int J Mol Sci. 2025 May 20;26(10):4903. doi: 10.3390/ijms26104903 (PMC12112502; doi:10.3390/ijms26104903)
Supplement: Supplementary file 1 [file ijms-26-04903-s001.zip › ijms-3583576-supplementary.pdf]

Supplementary

A

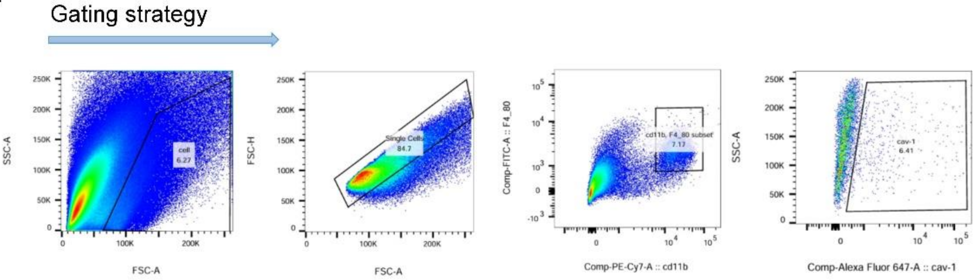

B

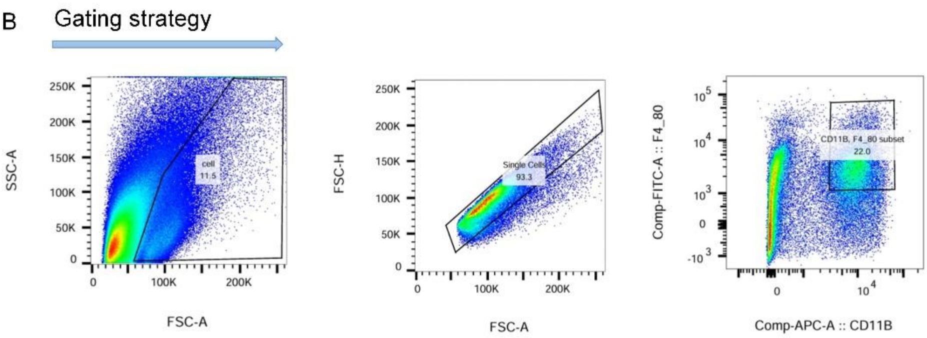

C

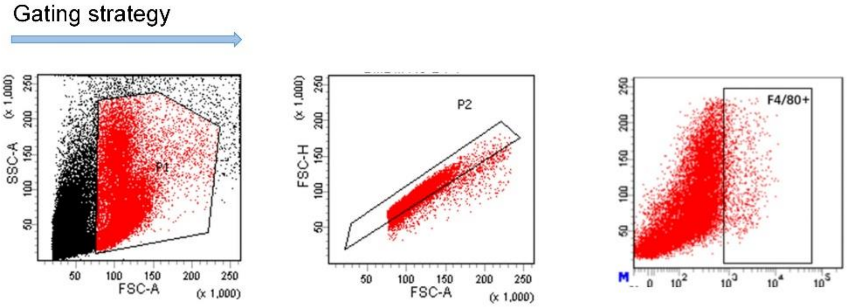

D

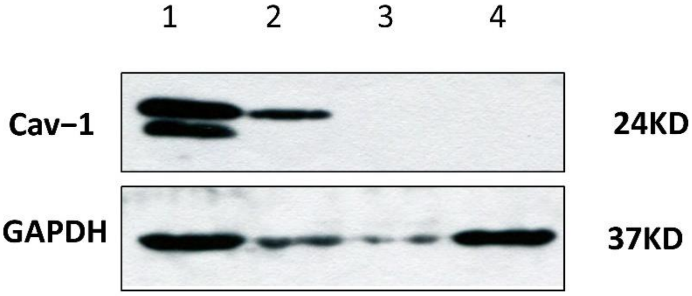

Supplementary

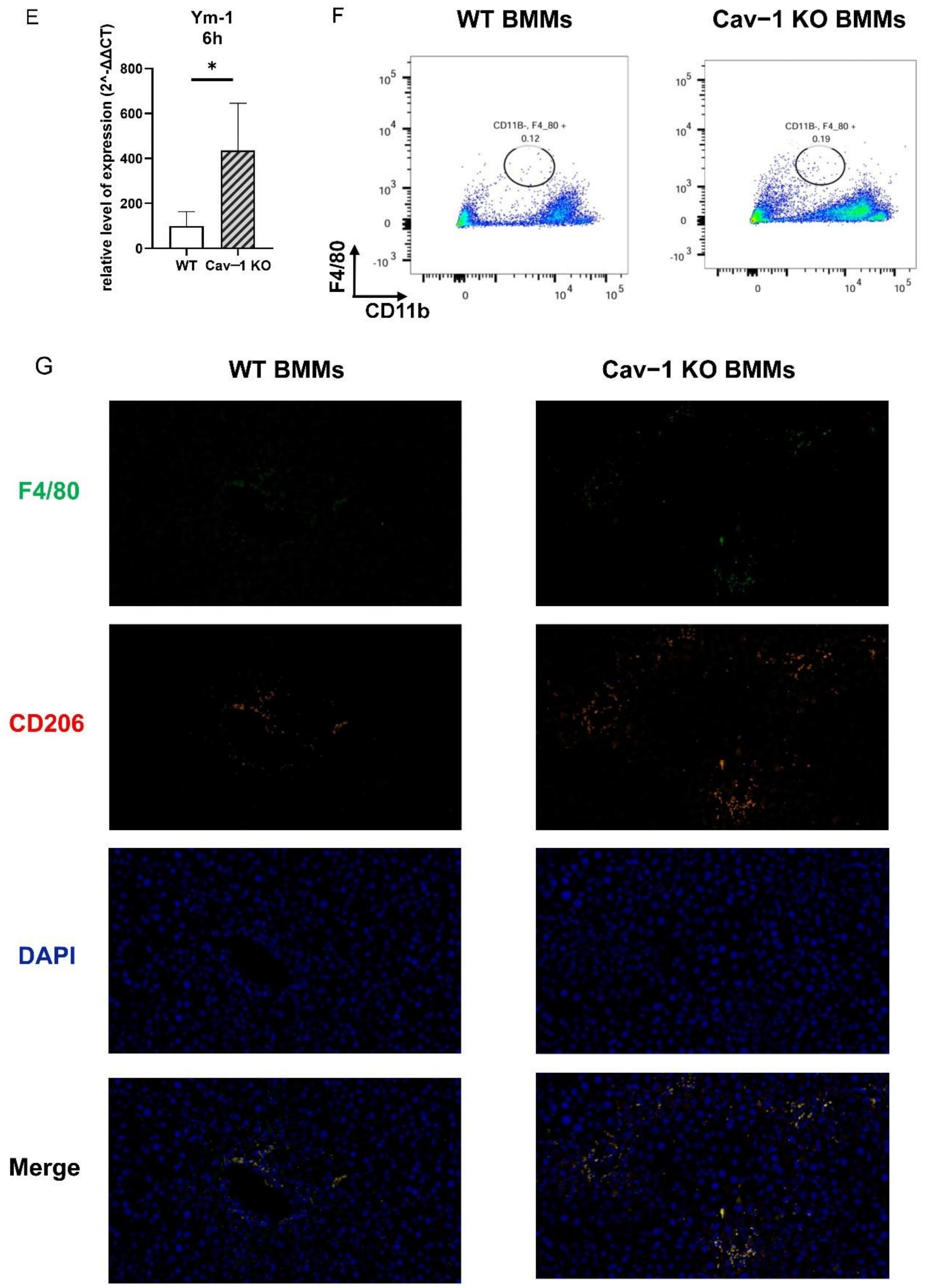

Figure S1

(A) In the analysis of acute liver injury models via flow cytometry, a gating strategy was employed to initially identify the CD11B+/F4/80+ cell population among the isolated hepatic non-parenchymal cells. Subsequent to this enrichment, the expression of Caveolin-1 within this macrophage subset was examined to elucidate its role in liver injury and repair processes (Fig

1A/B).

(B) Flow cytometry gating strategy for CCl4 injection 1week (Fig 2E).

(C) Flow cytometry gating strategy for macrophage clearance and reconstitution (Fig 7A).

(D) In our study, we utilized Caveolin-1 systemic knockout (KO) mice to investigate the role of Caveolin-1 in acute liver injured. To confirm the knockout efficiency of the Caveolin-1 KO mice, we performed Western blot analysis on protein extracts obtained from heart, liver, and lung tissues. This analysis was crucial to ensure the absence of Caveolin-1 protein expression in these organs, thereby validating the genetic modification in our mouse model. The WB analysis provided a definitive assessment of the KO efficiency, which is foundational for the interpretation of subsequent experimental outcomes.

1: WT mice lung;2: WT mice liver;3: Cav-1 KO mice lung;4: Cav-1 KO mice liver;

(E) Ym-1 mRNA expression in IL-4-treated BMMs (6 h).

(F) Representative flow cytometry plots for F4/80^{+}CD11B^{-} macrophages in damaged livers after Cav-1 KO or WT BMMs administration for 24 hours.

(G) Representative immunofluorescence images for F4/80+/CD206+ macrophages(green) in damaged livers after Cav-1 KO or WT BMMs administration for 24 hours.
